# Acupuncture for lumbar myofascial pain Protocol for a systematic review of randomized controlled trials

**DOI:** 10.1097/MD.0000000000016271

**Published:** 2019-06-28

**Authors:** Yupei Chen, Xiaohong Li, Jing Xu, Jie Chen, Zejun Huo, Li Zhang

**Affiliations:** a School of Acupuncture-Moxibustion and Tuina, Beijing University of Chinese Medicine; b School of Life Sciences, Beijing University of Chinese Medicine; c Department of Chinese Medicine, Peking University 3rd Hospital, Beijing, China.

**Keywords:** acupuncture, lumbar myofascial pain, protocol, systematic review

## Abstract

**Background:**

Lumbar myofascial pain is a major contributor to chronic low back pain. Acupuncture has been applied as an effective treatment for chronic low back pain worldwide. However, few critical systematic reviews focus on the effect of acupuncture on chronic low back pain caused by lumbar myofascial pain have been published. The study aims to evaluate the current evidence on the efficacy of acupuncture for chronic low back pain caused by lumbar myofascial pain.

**Methods:**

A total of 7 databases will be searched from their inception to March 2019, including PubMed, Medline, Embase, the Cochrane Central Register of Controlled Trials, the Chinese National Knowledge Infrastructure database, the Chinese Biomedical database, and the Wanfang database. Randomized controlled trials (RCTs) that compared the effect of acupuncture for lumbar myofascial pain will be included. The primary outcomes will be reduction of lumbar myofascial pain assessed by Visual Analog Scale (VAS). Secondary outcomes are questionnaires to evaluate the effects of treatment on patients’ daily life activities and psychological status; and adverse events. The primary and secondary outcomes will be assessed before (0 day) treatment and at 0, 7, 30, and 90 days after treatment. Data synthesis will be computed by RevManV.5.3.5 software when a data-analysis is allowed. Methodological quality will be evaluated with the risk of bias according to Cochrane Handbook.

**Results:**

The results of this study will be published in a peer-reviewed journal.

**Conclusion:**

The study will provide evidence to illustrate acupuncture is an effective therapeutic intervention for chronic low back pain caused by lumbar myofascial pain.

**Trial registration number:**

PROSPERO CRD42019129735

## 1 Introduction

Lumbar myofascial pain (LMP) is one of the important contributor to chronic low back pain (CLBP) that is associated with lots of adverse consequences, such as reduced quality of life, fatigue, limitations in activity of daily life, waste of medical resources, bad mood, and even disability.^[1–3]^ As the pain generator, myofascial trigger points (MTrPs) located in the corresponding lumber muscles are the pathological feature and treatment targets of LMP.^[4]^ Managing the pain origin from MTrPs could decrease LMP and play a significant role in alleviating CLBP.

There are many approaches have been reported to treat MTrPs such as traditional acupuncture,^[5–7]^ manipulation or massage,^[8]^ medications,^[9,10]^ dry needle,^[11–15]^ and other methods.^[16–18]^ Acupuncture has been applied for CLBP worldwide, however, it effect for CLBP caused by LMP has not been determined yet. So, we conducted this review to evaluate the effect of acupuncture for CLBP caused by LMP.

## 2 Methods

This review protocol has been registered on PROSPERO with number CRD42019129735 (http://www.crd.york.ac.uk/PROSPERO/display_record.php?ID=CRD42019129735). Cochrane Handbook for Systematic Reviews of Interventions and the Preferred Reporting Items for Systematic Reviews and Meta-Analysis Protocol (PRISMA-P) statement guidelines strictly comply in the protocol.^[19]^ Any change of the review will be described if needed.

### 2.1 Eligibility criteria

#### 2.1.1 Types of study

All clinical randomized controlled trials (RCTs) that label acupuncture for CLBP caused by LMP will be included without any language or publication status restrictions. Quasi-randomized trials and trials that cannot be ascertained if the trial is truly randomized will be excluded.

#### 2.1.2 Types of patients

##### 2.1.2.1 Inclusion criteria

1. Patients aged from 18 to 70 with a history of CLBP caused by LMP and duration of LMP is more than 6 months;
2. MTrPs were located at corresponding lumber muscles, such as quadratus lumborum muscle;
3. Mechanical stimulation on MTrPs inducing intense local and referred pain that is different from the pain expected on the basis of nerve root compression alone and often accompanied by withdrawal of the stimulated muscle.

##### 2.1.2.2 Exclusion criteria

1. Patients with any previous low back surgery, spondylolisthesis, facet joints arthropathy, and any other disorders about skeletal muscles system;
2. Patients received any acupuncture therapy that allows the muscular insertion and stimulation of needles within 6 month.

#### 2.1.3 Types of interventions

##### 2.1.3.1 Experimental interventions

The types of acupuncture included traditional acupuncture, manual acupuncture, electro-acupuncture, and dry needling. The research excluded trials testing acupuncture that used non-penetrating point stimulation; e.g., acupressure, massage, transcutaneous electrical nerve stimulation, magnets, and ultrasound therapy. Trigger point injection with any medication is excluded. The information of treatment cycle, frequency and follow-up has no limitation.

##### 2.1.3.2 Control interventions

The controls treatment could be sham acupuncture, no treatment, placebo acupuncture, acupressure, massage, medication, trigger point injection with medication. Studies compared acupuncture plus another therapy with the same other therapy alone will be included. Trials that only involve comparisons between different types of acupuncture are excluded.

#### 2.1.4 Types of outcomes

##### 2.1.4.1 Primary outcome(s)

Reduction of myofascial pain assessed by Visual Analogue Scale (VAS).

##### 2.1.4.2 Secondary outcome(s)

1. Questionnaires to evaluate the effects of treatment on patients’ daily life activities and psychological status.
2. Adverse events.

The primary and secondary outcomes were assessed before (0 day) treatment and at 0, 7, 30, and 90 days after treatment.

### 2.2 Search methods for the identification of studies

We will systematically search PubMed, Medline, Embase, the Cochrane Central Register of Controlled Trials, the Chinese National Knowledge Infrastructure database, the Chinese Biomedical database, and the Wanfang database from their inception to March 2019 without language restrictions. Search strategy of PubMed was as follows: “traditional acupuncture” or “manual acupuncture” or “electro-acupuncture” or “dry needle” and “lumbar myofascial pain” or “myofascial low back pain” or“lumber trigger points” or “low back myofascial trigger points” and“Randomized Controlled Trial” or “Randomized Controlled Trials as Topic” or “Controlled Clinical Trial”, Pin Yin (including “Zhen Ci” or “Zhen Jiu” or “Hao Zhen” or “Dian Zhen” or “Gan Zhen” or “Shou Zhen” and “Yao Ji Jin Mo Yan” or “Yao Bei Ji Jin Mo Yan” or “Yao Ji Ban Ji Dian” in the title and keywords sections).

### 2.3 Data collection and analysis

#### 2.3.1 Selection of studies

**Figure 1:**
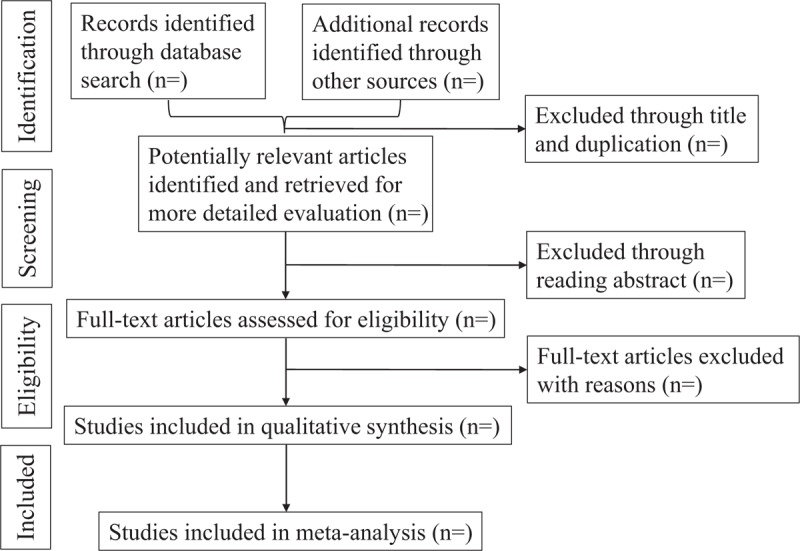
Flow diagram of study selection process.

All the authors involved in this study had previous experience of completing systematic reviews. We will use EndNote X8 to manage citations from databases. The title and abstract of each citation retrieved will be checked by 2 independent reviewers (CYP and LXH) according to eligibility criteria. The full texts of potentially relevant studies will be retrieved for further assessment. Disagreements will be solved by discussion of a 3rd author (ZL). A table named “reasons for excluded studies” will be established for the excluded studies. Details of the selection procedure for studies are shown in a PRISMA flow chart (Fig. 1).

#### 2.3.2 Data extraction and management

We will use predefined extraction forms with detailed written instruction which will be created using Microsoft Excel 2017 to collect relevant information and data. The information will include first author, year of publication, sample size, participants, interventions, sites of MTrPs, outcomes, and adverse events. The results of the data extraction will be checked by a senior reviewer (ZL). When the data of articles are sufficient or ambiguous, we will contact the corresponding authors for more information by e-mail or telephone.

#### 2.3.3 Risk of bias of included studies

Two of reviewers independently used the Cochrane Handbooks version 5.1.017 for systematic reviews of intervention to assess the quality of included RCTs,^[19]^ which includes 6 dimensions: adequate sequence generation, allocation concealment, blinding, the presence of incomplete data, selective reporting, other forms of bias. We will evaluate methodological quality as low, high, or unclear risk of bias.

#### 2.3.4 Measures of treatment effect

Outcome data were summarized using a risk ratio (RR) with 95% confidence intervals (CIs) for binary outcomes or mean differences (MDs) with a 95% CI for continuous outcomes.

#### 2.3.5 Unit of analysis issue

Considering that some studies compared 2 or more intervention groups with a control group, the research team followed the recommended advice in the Cochrane Handbook version 5.1.017 and combined groups to create a single pair wise comparison to avoid a unit-of-analysis error.

#### 2.3.6 Dealing with missing data

We will try to contact the first author by e-mail or telephone to obtain the missing data if possible. If failed, we will analyze the available data and discuss the potential influence of the missing data in the discussion.

#### 2.3.7 Assessment of heterogeneity

Heterogeneity will be assessed with a standard x2 text according to the guideline of Cochrane Handbook. When the *I*^2^ value is <50%, Study will be regarded as no statistical heterogeneity if *I*^2^ value is <50%, and the fixed-effect model will be selected. It will be considered significant heterogeneity while *I*^2^ ≥ 50%, and we will select a random-effect model and make subgroup analysis to explore the potential causes of heterogeneity.

#### 2.3.8 Data synthesis and analysis

Reviewer Manager Software (RevMan 5.3.5) from Cochrane Collaboration was used for data synthesis and analysis. A random-effects will be used when *I*^2^ ≥ 50%. When significant clinical heterogeneity existed, we will use subgroup analysis or sensitivity analysis, or only descriptive analysis.

#### 2.3.9 Assessment of publication bias

A funnel plot analysis was conducted to determine publication bias if 10 or more studies are in the meta-analysis.

#### 2.3.10 Subgroup analysis

Subgroup analyses were performed for type of acupuncture if at least 2 trials were available for which *I*^2^ > 50%.

#### 2.3.11 Sensitivity analysis

Sensitivity analysis will be performed to identify the robustness of studies according to the following criteria: methodological quality, sample size, and missing data.

#### 2.3.12 Quality of evidence

The quality of evidence for the primary outcomes will be assessed using Grading of Recommendations Assessment, Development, and Evaluation (GRADE),^[20]^ according to the comprehensive result of factors (risk of bias, inaccuracy, inconsistency, indirectness, publication bias) that influenced evidence quality which grades 4 levels: high level, moderate level, low level, and very low level.

## 3 Discussion

LMP, generated from MTrPs, is a major cause for CLBP. Disrupting MTrPs is the way to alleviate LMP and CLBP.^[21]^ Acupuncture that targets MTrPs has been reported effective treatment for myofascial pain,^[22,23]^ so this maybe a good choice for clinicians to use when they encountered a LMP patient with MTrPs.

The therapeutic effect of acupuncture for CLBP caused by LMP has not been reported yet. Although a previous Cochrane review had assessed the effectiveness of dry needling of myofascial MTrPs associated with low back pain in 2017, however, dry needling just a small part of acupuncture science,^[24]^ and there have been some new RCTs involving CLBP caused by LMP after 2017, so it is necessary to make a new review. We hope this systematic review will provide evidence that acupuncture is an effective method for CLBP caused by LMP

## Author contributions

Yupei Chen and Xiaohong Li contributed to the conception of the study. The manuscript of the protocol was drafted by Yupei Chen and was revised by Li Zhang and Zejun Huo. The search strategy was developed by Jie Chen and run by Jing Xu, who will also independently screen the potential studies, extract data of included studies, assess the risk of bias, and finish data synthesis. Yupei Chen and Xiaohong Li will arbitrate the disagreements and ensure that no errors occur during the study. All authors have approved the publication of the protocol.

**Conceptualization:** Yupei Chen, Xiaohong Li.

**Data curation:** Yupei Chen, Xiaohong Li, Jing Xu, Jie Chen.

**Formal analysis:** Jing Xu, Jie Chen.

**Funding acquisition:** Zejun Huo.

**Resources:** Yupei Chen, Xiaohong Li.

**Writing – original draft:** Yupei Chen.

**Writing – review & editing:** Zejun Huo, Li Zhang.

Yupei Chen orcid: 0000-0002-8146-3159.
